# Modelling energy metabolism dysregulations in neuromuscular diseases: A case study of calpainopathy

**DOI:** 10.1016/j.heliyon.2024.e40918

**Published:** 2024-12-09

**Authors:** Camille Siharath, Olivier Biondi, Sabine Peres

**Affiliations:** a Laboratoire de Biométrie et de Biologie Évolutive, UMR CNRS 5558 Université Claude Bernard Lyon 1, 69622, Villeurbanne cedex, France; b ERABLE, INRIA Lyon Centre, 69622, Villeurbanne cedex, France; c Laboratoire de Biologie de l'Exercice pour la Performance et la Santé (LBEPS), UMR, Université d'Evry, IRBA, Université de Paris Saclay, 91025, Evry-Courcouronnes, France

## Abstract

Biological modelling helps understanding complex processes, like energy metabolism, by predicting pathway compensations and equilibrium under given conditions. When deciphering metabolic adaptations, traditional experiments face challenges due to numerous enzymatic activities, needing modelling to anticipate pathway behaviours and orientate research. This paper aims to implement a constraint-based modelling method of muscular energy metabolism, adaptable to individual situations, energy demands, and complex disease-specific metabolic alterations like muscular dystrophy calpainopathy. Our calpainopathy-like model not only confirms the ATP production defect under increasing energy demands, but suggests compensatory mechanisms through anaerobic glycolysis. However, excessive glycolysis indicates a need to enhance mitochondrial respiration, preventing excess lactate production common in several diseases. Our model suggests that moderate-intensity physiotherapy, known to improve aerobic performance and anaerobic buffering, combined with increased carbohydrate and amino acid sources, could be a potent therapeutic approach for calpainopathy.

## Graphical abstract

**Figure:**
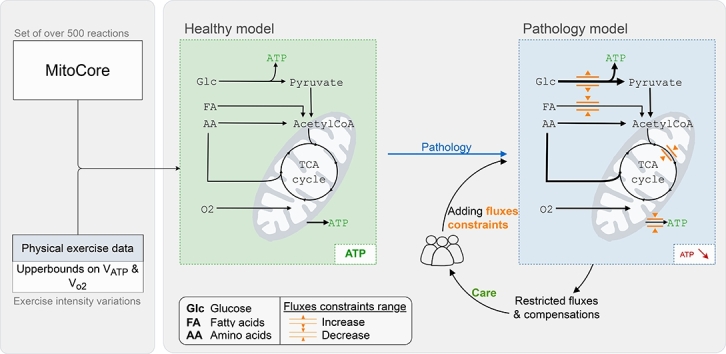

## 1 Introduction

At the core of living tissue, the cell serves as a functional unit [1], coordinating a vast range of biochemical processes essential to life. This entity harbours a dynamic world of subcellular structures and molecular mechanisms, within which energy metabolism operates. It defines the set of pathways, known as metabolic pathways, corresponding to a complex network of enzymatic reactions necessary for the renewal of the cellular needed energy, continuously changing according to the demand [2], [3].

Within these metabolic pathways, glucose degradation initiates within the cytoplasm, leading to the synthesis of adenosine triphosphate (ATP) and pyruvate, subsequently diverging into lactate or acetyl-CoA depending on cellular demand. Acetyl-CoA, which is also produced from fatty acids via the *β*-oxidation pathway and from the breakdown of amino acids, plays a crucial role in the tricarboxylic acid cycle, facilitating energy production in the mitochondria.

Among the possible known factors impacting energy metabolism, physical exercise causes major changes [4], [5], requiring the body to adapt to supply the energy needed for muscular contraction. Depending on the intensity, duration, and type of exercise, the amount and rate of energy consumption changes. This leads to the activation of complementary pathways, and the tune control of the enzymatic activities through cross-reaction between the different metabolites of the cells as well as of the organs.

In this context, fatty acids, through the *β*-oxidation, represent the most efficient cellular energy source but are constrained by production rates. Increased energy demand leads cells to resort to alternative sources, such as more powerful sources like glucose. Interestingly, because all of the energy pathways are interconnected and finely regulated by common co-activators and co-inhibitors, the overuse of glycolysis and carbohydrate oxidation in case of high energy demands, will limit the use of fatty acids. Energy metabolism is, therefore, a complex system and it is difficult to predict its behaviour simply by knowing its elementary components. It is therefore necessary to model it to facilitate its understanding and study [6].

Health condition is also a major factor influencing changes in energy metabolism, notably by affecting the way the body produces, stores, and uses energy. Multiple chronic and inherited diseases, such as cancer, diabetes, and cardiovascular diseases, lead to metabolic alterations, particularly in the mitochondria, which can participate in the disease progression by limiting cell functioning and/or favouring inflammation and pathogenic functions. Among these diseases, neuromuscular diseases represent a critical clinical challenge by their multiplicities in terms of origin and symptoms. While metabolic defaults can be a direct consequence of disease, certain neuromuscular disorders, such as calpainopathy, present metabolic defaults as a secondary effect, making it even more challenging to understand and possibly anticipate. Given the involvement of various enzymes, traditional experimental approaches would face limitations as it would be complicated to test each enzyme. Therefore, the need to develop new tools aimed at modelling the energy metabolism and its adaptation to different contexts of demand is of paramount importance and could be used as a lever for the development of new therapeutic approaches.

Calpainopathy [7], also known as Limb Girdle Muscular Dystrophy type 2A (LGMDR1), is an autosomal recessive genetic disorder affecting the proximal muscles of the shoulder and pelvic girdles. It is characterised by muscular atrophy leading to a progressive loss of mobility in affected patients. Unlike other dystrophies, calpainopathy is caused by mutations in the *CAPN3* gene, which codes for a calcium-dependent protease, calpain-3, involved in calcium homeostasis, and in the remodelling of sarcomeres, the multiprotein structures responsible for contraction. In the case of muscle damage, persistent calcium influx activates calpain-3, enabling the muscles to repair and adapt. Mutation of the *CAPN3* gene induces a loss of expression of the protein, leading to a disorganisation of sarcomeres, a deregulation of calcium flux and, as a secondary consequence, oxidative stress and energy metabolic alterations. Among these alterations, the loss of calpain-3 leads to the dysfunction of mitochondria, the alteration of *β*-oxidation, and a loss of equilibrium between the different energy pathways ultimately leading to a decline in ATP production rate [8], [9]. While the exact mechanisms by which this occurs are not yet fully understood, the understanding of its exact disturbance among the disease heterogeneity is of definite interest at preclinical and clinical levels.

To this date, there is no cure for this disease, and clinical care strategies remain based on symptom and pain relief. With the idea of studying the potential of physical exercise as a complementary approach to improve the clinical care of patients with this disease, previous research made the hypothesis that an exercise protocol, tailored to each patient's metabolic state, could lead to improvements in motor function and be considered as a clinical care strategy [5], [10].

Modelling in biology is usually used to decipher biological processes, to reproduce experimental results or, conversely, to predict experimentally unstudied events. In the case of metabolism studies, we can predict which pathway will be favoured by given conditions (initial conditions of metabolites, pathways voluntarily blocked) or, on the contrary, which pathways will be altered by changes in enzymatic activity. The main powerful methods for analysing and predicting the properties of genome-scale metabolic models are Flux Balance Analysis (FBA) [11] and related approaches [12], [13], [14]. The fundamental assumptions underlying the FBA framework for computing solutions are the steady state condition and the principle of optimality. The goal of FBA is to identify a flux distribution that achieves the maximisation of an objective function, such as the rate of biomass production or energy production.

An approach to enhance the quantitative accuracy of FBA predictions involves imposing constraints on uptake fluxes. In absence of measurements of these fluxes, FBA computations depend on setting bounds for exchange reactions to simulate specific cellular environments. Various techniques have been employed to approximate uptake fluxes, leveraging intricate details of regulatory networks [15], transporter saturation kinetics [16], transcriptomic data [17], and training datasets for machine learning-based methodologies [18].

We chose to use MitoCore [19], a constraint-based metabolic model specifically designed to capture mitochondrial processes. Constraint-based models such as MitoCore work by imposing physiological constraints to simulate steady-state metabolic fluxes. This approach enables us to anticipate key aspects of energy metabolism in healthy and pathological conditions, and to understand how cells balance pathways such as oxidative phosphorylation and glycolysis. MitoCore's focus on mitochondrial pathways makes it particularly useful for understanding energy dysregulation, specifically in diseases such as calpainopathy where mitochondrial dysfunction plays a central role. In addition, the model captures reversible reactions, such as those catalysed by enzymes like lactate dehydrogenase, enabling us to examine changes in metabolic fluxes under variable cellular conditions.

While not dynamic in the traditional sense, constraint-based modelling allows for a snapshot of the metabolic state under specific conditions such as those defined by energy demand, substrate availability, and environmental factors (nutrient intake) and the addition of constraints allows us to mimic energy production for multiple energy demands and its defects with altered metabolic pathways instead of blocked ones.

It is in this context, at the interface between physiology, pathophysiology and metabolic modelling, that this study takes place. Using Flux Balance Analysis (FBA) [11], we have constructed a healthy constraint-based model of the energy metabolism of a murine muscle cell that we can adapt to different pathological or individual situations, providing an instantaneous view of metabolic fluxes. First, we simulated on this model the multiple energy demands of physical exercise, expressed at 4 different intensities of maximum oxygen consumption, called $VO2_{max}$. These intensities correspond to the extremes of energy demand from rest (25%) to 100% of $VO2_{max}$, and including the intensities of the transitions between pathways: 65%, corresponding exclusively to the use of the aerobic pathway, and 85% corresponding to a combination of the aerobic and anaerobic pathways. This allowed us to ensure that the healthy model had a proper adaptation in the balance between the main energy pathways. Secondly, as a case study, we constrained this model with the known metabolic disorder in the most common limb-girdle dystrophy, calpainopathy. By applying predetermined values of key fluxes, we can compare the healthy to the calpainopathy-like model and highlight potential compensatory pathways and key regulators of the altered metabolism. The method developed in this study is intended to be adapted to other diseases with dysregulation of energy metabolism and should open up new potential therapeutic approaches.

## 2 Results

### 2.1 Healthy muscle cell's metabolic model under different energetic demands

In order to model muscle energy metabolism and to adapt it to different contractual conditions while allowing to anticipate compensatory mechanisms and/or potent modulators, we decided to adapt the well described MitoCore model [19], a mitochondrial metabolic model containing 485 reactions, including 155 mitochondrial reactions, 168 cytosolic reactions and 162 transport reactions of a human cardiac cell, to a healthy murine muscle cell used as control. To do this, we automatically checked for the presence of murine enzymes using the Brenda [20] and Unitprot [21] databases, suppressing only one enzyme for which no clear information supporting its existence in murine were found (see Method for more details).

Since the model relies on FBA with an objective function focused on biomass production, the fluxes of nutrient uptakes have to be settled. The original model has been defined with human cardiac glucose, fatty acids and amino acids fluxes entry, which do not correspond to murine skeletal muscle cell. Since these fluxes of nutrient uptakes, notably the glucose and fatty acids, are not well quantified for the murine skeletal muscle cell, we attempted to predict them using a proper FBA combined with Flux Variability Analysis (FVA) [22] approach, which optimises energy production with respect to the proportions of muscle energy pathways known from the literature [23] (see Method for more details), using experimental data of ATP production [23] associated to oxygen consumption [24] as constraints.

Under resting conditions (25% of maximal oxygen consumption) we obtained ranges of glucose and fatty acid fluxes allowing us to determine the exact value for each nutrient flux matching the expected proportion of 40% of carbohydrate oxidation (CHO) and 60% of *β*-oxidation. We then performed the same calculation but at different levels of exercise intensity with increasing ATP production rate, dioxygen consumption rate [24] and different proportions of energy pathways [23]. At 65, 85 and 100% of maximum oxygen consumption, FVA provides ranges of fluxes for each nutrient allowing us to determine these optimal values for both glucose and fatty acids (Table 1).

**Table 1:** Fixed constraint from experimental data of *O*_2_ inputs and ATP outputs fluxes set to compute the input values of glucose and fatty acids in the control model, and expressed in *μmol.gDW*^−1^.*min*^−1^. Each set of *O*_2_ and ATP is used to simulate 4 conditions of energy demands, expressed as 4 percentages of maximum oxygen consumption: 25, 65, 85 and 100% of *VO*2_*max*_. Glucose and fatty acids uptake fluxes were computed through a FVA method, allowing to respect constraints of energy demand, associated to carbohydrate oxidation (CHO) and *β*-oxidation proportions physiologically consistent [23].

Intensity of exercise (% of $VO2_{max}$)		25%	65%	85%	100%
Fluxes fixed	*O*_2_	-3.7	-7.9	-11.1	-14
	ATP	14.6	36.52	54.8	75

Fluxes computed by FBA	Glucose	-0.03	-0.13	-0.6	-2
	Fatty acids	-0.1	-0.27	-0.3	-0.08

**Figure 1:**
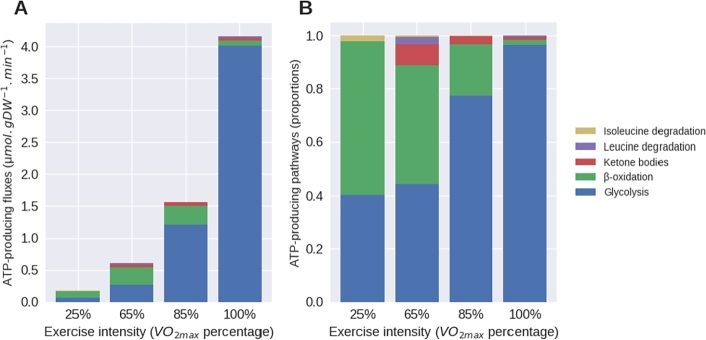
Fluxes expressed in absolute value (A) and proportions (B) of selected key enzymatic reactions from the main metabolic pathways used for ATP production, for each energy demand of physical exercise in a control muscle cell model. The reactions chosen as representative are phosphoglycerate mutase for glycolysis, acetyl-CoA acyltransferase for *β*-oxidation, 3-hydroxybutyrate dehydrogenase for ketone body degradation and leucine and isoleucine transaminase. The computed proportion of the main metabolic pathways are physiologically consistent, validating the behaviour of the control model.

This approach allowed us to determine that the needed fluxes of glucose in a single cell increase continuously from 25% to 100% of $VO2_{max}$ (Table 1) reaching a fold of 66. However, concerning the fatty acids entry in a cell, we noted an increase by 3 fold from 25% to 85% of $VO2_{max}$ then followed by a limitation of fatty acids entry due to excess of glucose consumption. Once the constraints on O2 consumption, ATP production, glucose and fatty acids had been applied, the fluxes obtained in the FBA solution had to be checked to validate the behaviour of our model. For this purpose, we selected key enzymatic reactions from the main energy pathways involved in the ATP production to evaluate the biological consistency of our model and the change in their balance with the intensity of energy demand (Fig. 1).

For each exercise intensity simulation, the instant fluxes (Fig. 1A) and proportions (Fig. 1B) of the ATP producing pathways changed as expected. We observed an increase by 57 fold in glycolysis flux from 25% to 100% of $VO2_{max}$. If the Isoleucine, Leucine and ketone bodies degradation was noticeable at 65% and 85%, their contribution to the total ATP produced dropped with the increase in intensity, whereas the fatty acids increased from 25% to 85% of $VO2_{max}$ and then totally dropped at 100% (Fig. 1A). Looking at the proportion (Fig. 1B) of metabolic pathways used to produce ATP, the results are consistent with the literature [23], and confirm that there is around 60% of fatty acids degradation (Fatty Acid metabolism) at rest, 50% at medium exercise intensity, 20% at high exercise intensity and around 2% at maximal intensity.

Thus, we have built and validated a control murine model of single-cell energy metabolism under multiple constraints of demands, which we can now adapt to any pathological condition.

### 2.2 Calpainopathy-like muscle cell's metabolic model under different energetic demands

To simulate the disease in the metabolic network model, we then gathered all reported mitochondrial consequences and metabolic dysfunctions associated with calpainopathy described in the literature from murine models [8], [9]. It is important to note that, to date the experimental data available are limited and only two studies provide quantitative or semi-quantitative evaluations of muscular metabolic alterations in calpainopathy. Most of these disease-induced regulations come from the analysis of differential gene expressions (RNAseq data) and few come from direct measurements of enzymatic activity. Regarding the studies reporting gene expression changes, the match between the list of enzymes in our control model with the RNAseq results gives us 18 altered enzymes of interest in the murine calpainopathy model (see Supplementary Table S4). These enzymes are mainly related to *β*-oxidation, the Krebs cycle, the mitochondrial respiratory chain, and amino acid degradation. Since it's known that a given amount of enzyme can give rise to different levels of activity depending on the cellular context and co-factors, we assumed that only the maximal enzymatic activity is proportional to the total gene expression. Concerning the data obtained from direct enzymatic activity measurements in the muscle of the calpainopathy murine model, only two enzymes from glycolysis matched with the enzyme included in our control model [8]. The activity of aldolase was reported to increase by 133%, while the activity of lactate dehydrogenase increased by 287% when compared to non-mutated animals. Because the reported enzymatic activities were measured under controlled conditions *in vitro*, we also assumed that these activities referred to their maximum. Thus, to build the calpainopathy-like model, we weighted the maximum fluxes calculated by FBA under all conditions of intensity from the control model for each selected enzyme, with their corresponding correction factor. The resulting fluxes are then used as an upper bound for the model, allowing variations in enzyme activity but limiting their maximum.

**Figure 2:**
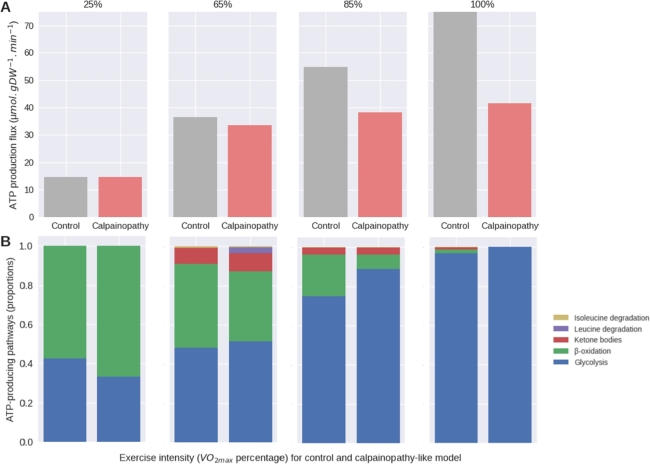
Comparison of the ATP production fluxes (A) and the proportions (B) of its main contributing pathways, for each intensity of energy demand, in control and calpainopathy-like models. The reactions chosen as representative are phosphoglycerate mutase for glycolysis, acetyl-CoA acyltransferase for *β*-oxidation, 3-hydroxybutyrate dehydrogenase for ketone bodies degradation, leucine and isoleucine transaminase and glutamate dehydrogenase.

Our developed calpainopathy-like model, with disease-specific constraints, was then run with FBA calculation using the same nutrient entry fluxes than those calculated for the control model. Finally, we compared the capacity of the calpainopathy-like model to produce ATP at each intensity of $VO2_{max}$ (Fig. 2A) and the balance between energy pathways (Fig. 2B) with the control model (Fig. 2).

In both models, the total flux of ATP production increased with intensity level, but less so in the calpainopathy-like model (Fig. 2A). Although the disease-like model was able to produce the necessary ATP at 25% of $VO2_{max}$, as effort increases, the model became more and more deficient in terms of ATP production, dividing the required ATP by 1.8 at 100% of $VO2_{max}$ (Fig. 2A). Associated with these intensity-induced decreases in ATP production, we noted an imbalance between the energy pathways when compared to the control model overall to the detriment of *β*-oxidation (FA metabolism)(Fig. 2B). If at 25% of $VO2_{max}$, we failed to observe any change in the proportion between the main energy pathways, we noted a marked decrease in *β*-oxidation compensated by an increase in ketone bodies and amino acid degradation at 65% of $VO2_{max}$ (Fig. 2B). However, With the increase in intensity until 85% and then 100% of $VO2_{max}$, the calpainopathy-like model induced a marked switch to the glycolysis when compared to the control model with the decrease and then disappearance of all other sources of fuel (Fig. 2B). Taken as a whole, our calpainopathy-like model highlights that, with an equivalent source of nutrients when compared to the control model, energetic metabolism is no longer able to produce large amounts of ATP at a proper rate despite a switch in energy pathways proportion, suggesting the need to improve the nutrient uptake to compensate.

**Figure 3:**
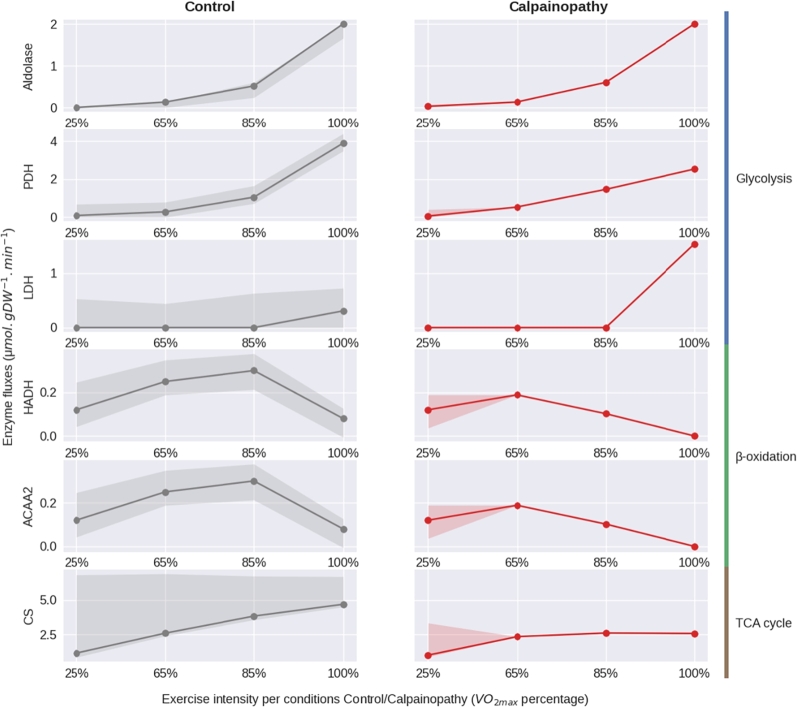
Plot of FBA solutions (dots) with the range of variability given by FVA solutions (blurred colour) for fructose biphosphate aldolase (aldolase, glycolysis), pyruvate dehydrogenase (PDH, glycolysis), lactate dehydrogenase (LDH, glycolysis), 3-hydroxyacyl-CoA dehydrogenase (HADH, *β*-oxidation), acetyl-CoA acyltransferase 2 (ACAA2, *β*-oxidation) and citrate synthase (CS, TCA cycle). Values are given according to the energy demand expressed as a percentage of maximal *O*_2_ consumption, in the control (left) and calpainopathy-like (right) models.

To better understand the disease-induced adaptation, we then ran a FVA analysis on selected enzymatic reactions from the main energy pathways at each intensity of energy demand that we plotted with the values of flux given by FBA results (Fig. 3). This allows us to evaluate the adaptability of the system and its capacity to modulate its flux level by showing the range of values that the flux of the enzyme of interest could take without changing the optimal value of the objective function (ATP production). This FVA-given range enables the solution to be adjusted and highlights possible compensations. Interestingly, our calpainopathy-like model showed a complete disappearance of any variability in enzymatic fluxes when compared to the control model from 65% of $VO2_{max}$ (Fig. 3), suggesting that under disease condition and common nutrient entry, we have only one solution to produce the highest amount of biomass. This unique solution per intensity highlighted an equal level of glycolysis between calpainopathy-like and control models over the different intensities in energy demand, noted by the aldolase flux, followed by a global limitation in oxidation power: limitation in TCA, *β*-oxidation, and PDH fluxes. In compensation, at 100% of $VO2_{max}$, the lactate production (lactate dehydrogenase) has increased from 0.3 to 1.5 $\mu mol.gDW^{-1}.min^{-1}$ in the calpainopathy-like model compared with the control model. This result suggests that the limitation of the oxidation capability of the model attempts to compensate through an increase in the anaerobic lactic metabolism but failed due to limited nutrient entry for a less powerful energy pathway. Taken as a whole, this modelling can not only anticipate the energy capabilities of a pathological condition, but can also highlight potent needed adaptations to compensate, leading to potential compensatory mechanisms to test.

### 2.3 Exploring compensation mechanism for ATP production in calpainopathy-like model

**Figure 4:**
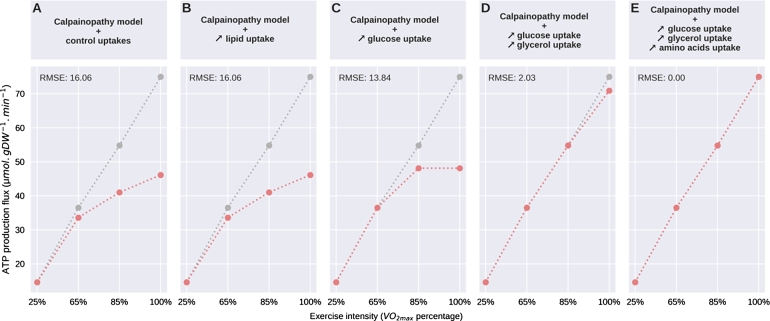
Plot of ATP production fluxes of the control model (grey dotted lines) compared to the calpainopathy-like model (red dotted lines) depending on the energy demand from 25 to 100% of *VO*2_*max*_. Evolution of the calpainopathy-like model fluxes of ATP production under control model nutrient uptake (A), by removing upper-bound of fatty acids (B), of glucose (C), of glucose + glycerol (D) and of glucose + glycerol + amino acids (E). The root mean square error (RMSE) is calculated to estimate the distance of computed values of calpainopathy-like model with those obtained with the control model.

Our pathological model relies on disease-induced enzymatic flux constraints and the set of nutrient influx upper-bounds. When we applied the same upper-bound of the different nutrients for the calpainopathy-like model as the control model, we observed a limitation in ATP production from 65% of $VO2_{max}$, without any glycolysis increase to compensate despite the elevation of aldolase upper-bound known in the literature. This suggests that the calpainopathy-like model could partly compensates its failure in producing energy by increasing its nutrient uptake. To demonstrate this hypothesis, we experimentally tested to remove the intake upper-bound of nutrients, nutrient per nutrient, for the calpainopathy-like model only, and we compared their capacity to compensate for the ATP production failure over each intensity of energy demand (Fig. 4). As expected, removing the upper-bound of fatty acids uptake didn't improve ATP production as the *β*-oxidation was already at its maximum capacity (Fig. 4B), with an identical Root Mean Square Error (RMSE) when compared to the control model than without removing the fatty acids upper-bound (Fig. 4A). However, if removing the upper-bound of ketone bodies didn't affect the ATP production (data not shown), removing them for glucose uptake allowed to fully correct the ATP production at 65% of VO2max, and enhanced the ATP production at higher intensity without matching the control model. This adaptation decreased the RMSE to 13 (Fig. 4C). When we removed upper-bounds of glucose and glycerol, we allowed the calpainopathy-like model to quietly fully recover the ATP production capacity, with RMSE at 2 (Fig. 4D), while adding the remove of amino acid upper-bound finished to correct the metabolic state (Fig. 4E). Our modelling suggests that despite enzymatic constraints, the calpainopathy-like model can compensate for its defect in ATP production through the combined increase of nutrient uptake, a new approach for this disease that should be evaluated *in vivo*.

**Figure 5:**
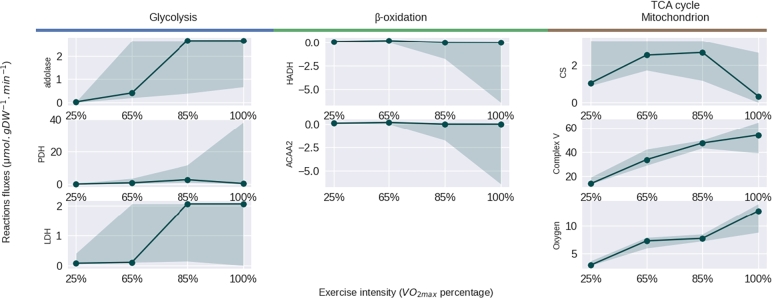
Plot of FBA solutions (dots) with the range of variability given by FVA solutions (blurred colour) for fructose biphosphate aldolase (aldolase, glycolysis), pyruvate dehydrogenase (PDH, glycolysis), lactate dehydrogenase (LDH, glycolysis), 3-hydroxyacyl-CoA dehydrogenase (HADH, *β*-oxidation), acetyl-CoA acyltransferase 2 (ACAA2, *β*-oxidation), citrate synthase (CS, TCA cycle), ATP synthase (complex V, TCA cycle) and oxygen transport (oxygen, TCA cycle) of the calpainopathy-like model when we fully restored the ATP production rate by removing the upper-bounds of glucose, glycerol and amino acids. Values are given according to the energy demand expressed as a percentage of maximal *O*_2_ consumption.

When we focus on this restored situation to better understand the underlying mechanisms ensuring this correction, we ran an FVA analysis on the main enzymes of interest (Fig. 5). The results showed that increasing the possibility of uptake, restored a possible variability for each flux. Confirming previous results and the necessity to increase other sources of energy, we can see here that glycolysis activity increases over time reaching its maximum activity illustrated by aldolase and lactate dehydrogenase, as well as the *β*-oxidation. Interestingly, not only enzyme activity of *β*-oxidation are limited by the regulation applied on the calpainopathy-like model, but the FVA results also show a change in reactions orientation meaning the model could produce fatty acids instead of oxidising them. When looking at the activity of the TCA cycle, we can see a decrease in citrate synthase activity from 85% of $VO2_{max}$, while oxygen transport and Complex V activities are still increasing, an behaviour enabled by the increase of amino acids uptake, allowing the production of ATP to reach its objectives.

## 3 Discussion

In this study, we built a constraint-based metabolic model of a murine healthy skeletal muscle under different energy demands that we can adapt to various pathological or individual situations. We adjusted the upper bounds of the uptake fluxes of main nutrients (glucose and fatty acids) to match the known evolution of the proportions of energy pathways with an increase in O2 consumption and ATP production mimicking a range from 25 to 100% of maximal O2 consumption. The values for ATP production and O2 consumption were extracted from experimental values, while we determined adequate nutrient uptake. Based on this correspondence between energy production and nutrient consumption, we have a model of muscular energy production ready to be adapted to disease contexts to evaluate metabolic changes and possibly anticipate compensatory mechanisms. To this end, we focused on calpainopathy disease, the most common limb-girdle muscular dystrophy, in which complex metabolic alterations have been reported as indirect defects of the *CAPN3* gene mutation. To model this pathology, we combined published dysregulations of muscle metabolism in a calpainopathy murine model and used these alterations to constrain enzyme fluxes by calculating dedicated bounds. The tune analysis of this calpainopathy-like model, not only confirmed a loss of ATP production capacity but also revealed for the first time that increasing the uptake of a combination of nutrients (glucose + glycerol + amino acids) can overcome the energy production defects. Our study not only developed mitochondrial bioinformatic models but also showed that we can use such models to orientate preclinical and clinical research, creating an adaptive tool for personalised medicine.

Our study is the first attempt to model dystrophic disease with secondary metabolic alterations using a constraint-based method. This unique approach allowed us to mimic *in vivo* energy production defects with altered metabolic pathways instead of blocked ones. The increase and decrease of enzymatic activity related to a specific disease context is of paramount challenge, knowing that for an equivalent enzymatic quantity, its activity can be multiplied under specific cellular contexts, such as a decrease in ATP content and/or a change in the amount of cofactors. This leads us to consider two priorities for the model: 1. a drastic change in energy demand to simulate changes in muscle fate under different conditions of use; 2. the use of FBA and FVA allowing a range of enzymatic fluxes. The combination of these two parameters opens new avenues for further research in the field. As a proof of concept of the interest of such an original approach, we used calpainopathy as a pathological context to constrain the model. Interestingly, thanks to the modelling of a gradual increase in energy demand, we were able to objectify the muscular defect in ATP production while at rest the modelled cell did not show any defect. Moreover, this approach also highlighted the imbalance between pathways that change for each percentage of energy demand, revealing the complexity of cellular adaptation and providing a precise guide for experimental research. However, of the three enzymes known to be up-regulated in calpainopathy, two were represented in the model (lactate dehydrogenase and fructose biphosphate aldolase), and only lactate dehydrogenase flux was used at its potential compared to the control at 100% of $VO2_{max}$ (see supplementary table S2), suggesting a compensatory overuse of the anaerobic lactic acid pathway in this disease context. The value of fructose-biphosphate aldolase flux, an enzyme that catalyses the first steps of glycolysis, remained the same as control at all levels of energy demand, consistent with fixed glucose uptake and thus limiting potential compensations. If this could highlight the limitations of the FBA method due to its dependence on fixed nutrient uptake, it also demonstrates its ability to focus on blocking steps of energy metabolism, opening up compensatory mechanisms to decipher. This hypothesis is also confirmed by the FVA analysis (Fig. 3), which shows little variability for the resting state and none for the other states with higher energy requirements. Disease-induced regulations we used to build the model, therefore allowed only one solution and no alternative pathway, meaning there is no possible adjustment with the first set of parameters fixed. The calpainopathy-like model is therefore a constrained model offering little to no flexibility compared to the control model. However, it's well known that the nutrient uptake capacity of a cell can vary under different conditions, pathological or under treatment or exercise training. Importantly, when we modulated the nutrients uptake fluxes of the model, to observe compensation and reduce the current ATP deficit, FVA showed a return of enzymatic flexibility allowing the diseased model to produce more ATP and to adapt differently to a change in ATP consumption rate. This result not only pushes the preclinical research to focus on muscle uptake capacity but also open avenues for therapeutic approaches to improve glucose uptake, including physiotherapy.

By first increasing the glucose and FA inputs, we were able to confirm that *β*-oxidation had already reached its maximum activity, as the increase in FAs didn't change the lack of ATP production, illustrating the inability of the muscle to trigger the expression of genes associated with fatty acid metabolism after exercise [8]. Since *β*-oxidation and the mitochondrion are limited by regulation, the model should be able to produce more ATP via the anaerobic-lactic pathway to compensate. This is indeed the case at all intensities, allowing greater ATP production than with the baseline glucose value and even reaching ATP demand at 65% of $VO2_{max}$. However, glycolysis is limited both by the constraints applied to fructose-biphosphate aldolase (maximum flux of 2.66 $\mu mol.gDW^{-1}.min^{-1}$) and by those applied to lactate dehydrogenase (maximum flux of 2.05 $\mu mol.gDW^{-1}.min^{-1}$), values that are reached for a glucose input flux of 3 $\mu mol.gDW^{-1}.min^{-1}$ at 85% and 100% of $VO2_{max}$.

It might be tempting at first to try to remove these limitations in order to improve the anaerobic lactic pathway. However, increasing lactate production may not be a viable solution, as several diseases are characterised by an excess of lactate production, which we generally try to avoid as it may lead to lactic acidosis [25]. So although these results showed a corrective potential through glycolysis, increasing glucose uptake flux alone is not enough to compensate for the lack of ATP production. Therefore, in addition to the variation in glucose, we also allowed variation for other energy sources by increasing their possible uptake. Increasing amino acid uptake alone allowed compensation, but their flux values were well above what could be found in the literature, with a 5 fold [26]. By combining ketone bodies, glycerol and amino acid uptake, we were able to address this problem and show ATP production compensation within a more consistent range. Our study has demonstrated the potential of multiple pathway combinations to compensate for disease alterations and to reinforce the interconnection of the metabolic pathways, especially in the context of mitochondrial dysfunction, a common feature of numerous neuromuscular disorders.

To characterise oxidative phosphorylation activity, we analysed key metrics such as the rate of oxygen consumption and ATP synthesis, which are essential indicators of mitochondrial function. Concerning the design of the control model and its functioning under gradual energy demands, knowing the ATP consumption rate over different O2 consumption rate was not sufficient to mimic physiological muscular function. The FBA-based model failed to produce the required ATP or balance the metabolic pathways at the published nutrient limits. As O2 and ATP consumption increases, muscle cells use different metabolic pathways according to their respective “power”, i.e. their respective ability to produce a certain amount of ATP per unit of time. If *β*-oxidation is defined as the most efficient pathway, the number of subsequent enzymatic steps to produce ATP slow down their capacity which then has to be compensated by glycolysis, less effective but much faster. Unfortunately, our model failed to mimic this physiological adaptation pushing us to search for proper nutrient boundaries ensuring the match between energy demand and the known proportions of the main metabolic pathways. This manual adjustment of the control model highlight FBA approach limitations which should be addressed by combining other computational calculation to properly anticipate the special cases we would model. To enhance our approach, integrating genome-scale metabolic models with transcriptomics data, facilitated by tools such as COBRApy, could offer a more comprehensive view of cellular metabolism. This integration would allow for more accurate predictions of metabolic fluxes, leading to better constraints on nutrient uptake rates and more reliable metabolic behaviour predictions. Recently, a machine learning method has been proposed [27] to learn and generalise statistical patterns between input conditions and output reference fluxes. However, these models still suffer from the lack of experimental data set to train. Moreover, it is worth noting that our method does not take into account the concentration of either the metabolites or the enzymes. Knowing the kinetic parameters could allow us to find more accurate uptake values, in particularly the glucose uptake, and to predict the variation of enzymatic activities for possible therapeutic targets. However, calculating the concentration of metabolites and enzymes in metabolic networks is a challenging task due to the non-linear nature of enzyme kinetics. When including non-linear enzyme kinetics into models that contain alternative pathways, the solution space is no longer convex, making such problems extremely difficult to solve computationally. One approach to address this challenge is the Enzyme Cost Minimization [28] algorithm, which identifies the set of metabolite concentrations that minimise the total enzyme mass required to sustain a pre-determined flux through a single pathway. To find the optimal pathway in metabolic networks [29], all elementary flux modes [30] (EFMs) must be computed. However, the number of EFMs increases combinatorially for large-scale networks. Even if the computation of relevant EFMs that respect diverse linear and boolean constraints is possible with a large set of constraints [31], the identification of all the kinetic parameters is challenging [32], [33].

To validate and refine our model predictions, several experimental approaches can be employed. One key method involves combining oximetry measurements with ATP production rate assessments on isolated mitochondria from human biopsies or animal model muscles [34]. Additionally, direct enzymatic activity measurements of key reactions in the muscular energy pathways of interest can be performed. For instance, assessing the activities of phosphofructokinase and hexokinase would provide valuable data on glycolytic flux. These experimental approaches would not only serve to validate our model's predictions but also provide new data to further refine and improve the model's accuracy. Furthermore, these experimental validations could be extended to compare healthy and calpainopathy-affected tissues, allowing us to verify the predicted metabolic alterations and potentially identify new therapeutic targets. By iteratively integrating experimental data and model predictions, we can develop a more comprehensive understanding of energy metabolism dysregulations in calpainopathy and potentially other neuromuscular diseases.

In conclusion, our pioneer approach intends to be a generic model of murine muscle metabolism, making it suitable for any pathology with available data on regulatory enzyme activities. Our method operates automatically, and can be continuously optimised with experimental enrichment of clinical data, pointing out metabolic blockage, compensatory mechanisms and opening the way to new therapeutic approach as well as to personalised medicine.

### 3.1 Limitation of the study

It is important to acknowledge that this study is based on data from only two studies, with different kind of cells, and in various kinds of experiments (RNAseq and enzymatic activities). This reflects the current scarcity of quantitative data on metabolic alterations in calpainopathy. Thus, the computed calpainopathy-like model may not reflect all cases of the different variations of the disease, but despite this limitation, our work serves as a prof-of-concept, showing how constraint-based modelling can integrate available experimental data to generate testable hypotheses about disease mechanisms and potential therapeutic approaches. As this is meant to be an upgradeable model, designed to guide the biology researcher, it can then easily be supplemented with new enzymatic activity, or expression data, by adding the type of regulation to be taken into account to the existing list. Therefore analyses were carried out in this study to validate similar behaviours of the metabolism of a calpainopathy muscle cell, but this approach could be adapted to other neuromuscular diseases, providing a valuable tool for understanding metabolic dysregulations and guiding therapeutic strategies.

## 4 Material and methods

This study was carried out using the MitoCore model [19], which was originally built to represent the metabolism of a human heart cell mitochondrion and therefore does not correspond exactly to the model needed. MitoCore includes 485 reactions covering all parts of central carbon metabolism.

### 4.1 Fitting MitoCore to murine metabolism

The first step to adjust this model to our study was to assure each reaction represented in MitoCore existed in mice metabolism. In order to do that, we took the KEGG ID associated to each reaction in our model, requested through the KEGG API the EC-number associated to the enzyme catalysing the reaction and checked the presence in murine in the BRENDA [20] database. In the set of reaction of MitoCore, 7 didn't get an entry in BRENDA and 39 didn't exist in murine, but after manually checking in literature, only one of them did not have clear evidence to support its existence, and was therefore removed of the model: the 4-acetamidobutyrate deacetylase.

### 4.2 Computation of carbon source allocation in muscle during exercise

In this study, we chose to build our healthy skeletal muscle cell model using only a constraint-based approach, without employing additional pipelines [35]. This decision allowed us to simulate the metabolic activity of skeletal muscle under varying energy demands, capturing key pathways such as oxidative phosphorylation and glycolysis. By applying generalised physiological constraints, we were able to explore core aspects of energy metabolism relevant to both healthy and disease conditions, without relying on tissue-specific omics data, which is required to build highly specialised models like those automatically generated. Several studies discuss the effects of physical exercise on skeletal muscle metabolism, but a smaller number include precise measurements of metabolite production and consumption [23], [36]. These measurements describe the flux of ATP produced and the proportion of pathways contributing to this production for different percentages of $VO2_{max}$. The proportions of glycolysis and fatty acids degradation expected for the healthy model are summarised in the Table 2.

**Table 2:** Requirements of metabolic pathway percentages between glucose and fatty acid sources, and sets of ATP production fluxes associated with *O*_2_ absorption fluxes for all energy demands of physical exercise. The fluxes are expressed in *μmol.gDW*^−1^.*min*^−1^. The literature-based constraints were used to run the FBA, while the pathways percentages were used to validate the model behaviour.

Intensity of exercise (percentage of $VO2_{max}$)		25%	65%	85%	100%
Pathways percentages	Glycolysis	40%	50%	80%	100%
	*β*-oxidation	60%	50%	20%	0%

Literature-based constraints	*O*_2_ absorption	-3.7	-7.9	-11.1	-14
	ATP production	14.6	36.52	54.8	75

Among the flux values of the absorbed metabolites, only oxygen values could be found [24], describing maximum oxygen consumption as a function of different exercise intensities in untrained rats. The values used as constraints on the models will therefore be those for oxygen consumption and ATP production also summarised in Table 2.

To determine the uptake of glucose and fatty acids for a given ATP production and O2 absorption respecting the proportions of carbohydrate oxidation (CHO) and *β*-oxidation in the Table 2, an optimisation problem has to be solved, computing a flux distribution for a maximal ATP production. We then computed the maximal uptake of glucose with FVA and decrease this value until the wanted proportions were reached. As the glucose uptake decreases, the fatty acids uptake increase to reach the maximal ATP production, allowing to find the right balance of pathways proportion.

### 4.3 Calpainopathy data

Metabolic dysfunctions were gathered from different studies. The first part of the regulations was measured on C57BL/6 mice [8], five of those consist of differential gene expression in plantaris muscle cells, between the control and the calpainopathy mice. For RNAseq data, it was assumed that only the maximal enzymatic activity was proportionally linked to the total gene expression to be used for model construction. Two regulations were taken from the same study but consist of frozen muscles glycolytic enzymes activities at 25 °C and particularly the difference of activity between the control and the calpain-3 knock-out cell.

The second part of the regulations was measured on immortalised muscle cell cultures from C57BL/6 mice grown at 33 °C and consisted of differential gene expression in myoblasts and myotubes. The temperature difference comes from the fact that the first study focused on measuring enzyme activities, while the second study set the temperature solely for cell growth, which did not influence gene expression.

And finally there are some reactions that are known to be subject to metabolic defaults such as the oxidation of fatty acids (Hydroxyacyl-Coenzyme A dehydrogenase) and their use for ATP production [8], and mitochondrial dysfunction. Although there are multiple cases regarding the activity of Citrate Synthase in calpainopathy (increase, decrease or no significant change), as mitochondrial dysfunctions are known to exist, a warning has been placed on the enzyme which translates as a limitation of its activity.

### 4.4 Solvers

The flux distributions to optimise energy production are carried out using Flux Balance Analysis (FBA) [11], [37], a mathematical approach that involves creating a mathematical model of the metabolic network and then using this model to predict the flux of metabolic molecules within the cell. Using COBRApy [38], an object-oriented Python library that provides the main COBRA methods and facilitates the representation of metabolic pathways and gene expression, the strategy is to maximise the ATP production, while constraining the dioxygen fluxes associated to each exercise intensity, and to infer glucose and fatty acid fluxes absorption that couldn't be found in the literature. The results relying on FBA solutions are deterministic regarding the constraints. Thus, repetition of the calculation always gives the same values for identical constraints, as optimal qualitative values.

In the case of multiple solutions that maximise the objective function, FBA offers one that may not be the one previously expected. To compute an alternative solution, Flux Variability Analysis (FVA) [22] is a method that provides a range of possible flux values for each reaction of the model, while maintaining the state of the network and thus satisfying the core optimisation problem of FBA. FVA offers a more complete view of the behaviour of the metabolic network, enabling variability and potential regulation points to be identified. The reaction bounds can then be modified accordingly to adjust the optimal solution, to approximate the characteristics of the different conditions studied.

## CRediT authorship contribution statement

**Camille Siharath:** Writing – original draft, Software, Methodology, Formal analysis, Conceptualization. **Olivier Biondi:** Writing – original draft, Methodology, Formal analysis, Conceptualization. **Sabine Peres:** Writing – original draft, Software, Methodology, Formal analysis, Conceptualization.

## Declaration of Competing Interest

The authors declare that they have no known competing financial interests or personal relationships that could have appeared to influence the work reported in this paper.

## Data Availability

This study was carried out in a conda [39] environment. The main focus of the article, which was to get parameters for the healthy mitochondrial muscle cell model and then to build a model simulating the energy metabolism of a calpainopathy case, was developed in Python, using the Cobrapy [38] and matplotlib libraries via a series of Notebooks [40] grouping together all the necessary research, construction and analysis steps. These notebooks use a Python library which itself contains a series of functions for building and displaying the results obtained. The notebook, script, model, other files and environment required for this study are all available on github (https://github.com/csiharath/mitocore_calpainopathy).

## Appendix A Supplementary material

MMC 1 Document S1. Tables S1-S3. S1 - List of key enzymatic reactions from the main energy pathways (Glycolysis, *β*-oxidation, TCA cycle) involved in ATP production to evaluate the biological consistency of our model. S2 - Fluxes of key enzymatic reactions computed through FBA for different levels of physical intensity in the control model, in *μmol.gDW*^−1^.*min*^−1^. S3 - Fluxes of key enzymatic reactions computed through FBA for different levels of physical intensity in the calpainopathy-like model, in *μmol.gDW*^−1^.*min*^−1^. MMC 1

MMC 2 MitoCore metabolic model adapted to a healthy murine metabolism in MATLAB format. MMC 2

MMC 3 MitoCore metabolic model adapted to a healthy murine metabolism in excel format. MMC 3

MMC 4 MitoCore metabolic model adapted to a healthy murine metabolism in SBML format. MMC 4

MMC 5 Calpainopathy-like muscle cell’s metabolic model in MATLAB format. MMC 5

MMC 6 Calpainopathy-like muscle cell’s metabolic model in excel format. MMC 6

MMC 7 Calpainopathy-like muscle cell’s metabolic model in SBML format. MMC 7
