# Supplementary material for: Modelling energy metabolism dysregulations in neuromuscular diseases: A case study of calpainopathy
Source: Heliyon. 2024 Dec 9;10(24):e40918. doi: 10.1016/j.heliyon.2024.e40918 (PMC11698924; doi:10.1016/j.heliyon.2024.e40918)
Supplement: MMC 1 — Document S1. Tables S1-S3. S1 - List of key enzymatic reactions from the main energy pathways (Glycolysis, β-oxidation, TCA cycle) involved in ATP production to evaluate the biological consistency of our model. S2 - Fluxes of key enzymatic reactions computed through FBA for different levels of physical intensity in the control model, in μmol.gDW−1.min−1. S3 - Fluxes of key enzymatic reactions computed through FBA for different levels of physical intensity in the calpainopathy-like model, in μmol.gDW−1.min−1. [file mmc1.pdf]

Supplemental information

Document S1. Tables S1-S3

Table S1: List of key enzymatic reactions from the main energy pathways (Glycolysis, β-oxidation, TCA cycle) involved in ATP production to evaluate the biological consistency of our model.

Enzyme	Associated reaction
**Glycolysis**	
**Fructose biphosphate-aldolase**	beta-D-Fructose 1,6-bisphosphate ↔ Glycerone phosphate + D-Glyceraldehyde 3-phosphate
**L-lactate dehydrogenase**	(S)-Lactate + NAD+ ↔ Pyruvate + NADH + H+
**β-oxidation**	
**3-hydroxyacyl-CoA dehydrogenase**	(S)-3-Hydroxydodecanoyl-CoA + NAD+↔ 3-Oxododecanoyl-CoA + NADH + H+
**Acetyl-CoA acyltransferase 2**	CoA + 3-Oxooctanoyl-CoA ↔ Hexanoyl-CoA + Acetyl-CoA
**TCA cycle**	
**Pyruvate dehydogenase**	Pyruvate + CoA + NAD+ ↔ Acetyl-CoA + CO2 + NADH + H+
**Citrate synthase**	Acetyl-CoA + H2O + Oxaloacetate ↔ Citrate + CoA
**Objective Function**	
**ATP hydrolysis**	H2O + ATP → ADP + Pi + H+ + Biomass
**Carrier**	
**O2 transport**	O2[externe] ↔ O2[cytosolique]

Table S2: Fluxes of key enzymatic reactions computed through FBA for different levels of physical intensity in the control model, in $\mu mol.gDW^{-1}.min^{-1}$

Intensity of exercise (percentage of $VO2_{max}$)	25%	65%	85%	100%
Fructose-biphosphate aldolase	0.03	0.13	0.60	2.00
Pyruvate dehydrogenase	0.11	0.20	1.30	3.84
L-lactate dehydrogenase	0.07	0.10	0.14	-0.39
3-hydroxyacyl-CoA dehydrogenase	0.10	0.27	0.30	0.08
Acetyl-CoA acyltransferase 2	0.10	0.27	0.30	0.08
Citrate synthase	1.10	2.80	3.76	4.69

Table S3: Fluxes of key enzymatic reactions computed through FBA for different levels of physical intensity in the calpainopathy-like model, in $\mu mol.gDW^{-1}.min^{-1}$

Intensity of exercise (percentage of $VO2_{max}$)	25%	65%	85%	100%
Fructose-biphosphate aldolase	0.03	0.13	0.60	2.00
Pyruvate dehydrogenase	0.03	0.42	1.34	2.53
L-lactate dehydrogenase	-0.07	-0.10	-0.14	-1.53
Citrate synthase	1.10	2.30	2.65	2.58
3-hydroxyacyl-CoA dehydrogenase	0.10	0.18	0.12	0.0
Acetyl-CoA acyltransferase 2	0.10	0.18	0.12	0.0
02 transport	3.25	6.75	7.78	7.81
ATP production	14.6	33.52	38.14	41.56

Table S4: List of calpainopathy regulations matching with our metabolic model, associated to a value which we applied as a factor on the maximal activity of the corresponding enzyme in order to calculate new bounds for the calpainopathy-like model.

Enzyme coding gene	Regulation
Isocitrate dehydrogenase (idh1)	1.69
Fructose-bisphosphatase (fbp2)	1.71
Alcohol dehydrogenase (adh1)	2.01
Oxoglutarate Dehydrogenase L (ogdhl)	2.05
Methylenetetrahydrofolate dehydrogenase (mthfd2)	2.18
Lactate dehydrogenase (LDHB)	2.83
Fructose biphosphate aldolase (aldoa)	1.33
A cyl-CoA synthetase short-chain family member (ACSS2)	2.5
3-oxoacid CoA transferase 1 (OXCT1)	0.65
NADH dehydrogenase (NDUFC1)	1.16995
Cytochrome c oxidase (COX7A2L)	0.55
ATP synthase (ATP5C1)	1.2123
Branched chain amino-acid transaminase 2 (BCAT2)	2.01
Multiple methyltransferases (EEF2)	2.29526
Cysteine dioxygenase type 1 (CDO1)	0.22
Aldehyde dehydrogenase 18 family, member A1 (ALDH18A1)	2.06886
Glutathione peroxidase (GPX3)	0.35
ATPase phospholipid transporting 8B1 (ATP8B1)	0.67
Cistrate synthase (CS)	Warning
Hydroxyacyl-CoA Dehydrogenase (HADH)	Warning
